# Diatom Biosilica Doped with Palladium(II) Chloride Nanoparticles as New Efficient Photocatalysts for Methyl Orange Degradation

**DOI:** 10.3390/ijms22136734

**Published:** 2021-06-23

**Authors:** Myroslav Sprynskyy, Paulina Szczyglewska, Izabela Wojtczak, Izabela Nowak, Andrzej Witkowski, Bogusław Buszewski, Agnieszka Feliczak-Guzik

**Affiliations:** 1 Faculty of Chemistry, Nicolaus Copernicus University in Toruń, 7 Gagarina Str, 87-100 Toruń, Poland; izabelawojtczak1991@gmail.com (I.W.); bbusz@chem.umk.pl (B.B.); 2 Department of Biopharmaceutics and Pharmacodynamics, Medical University of Gdańsk, Hallera 107, 80-416 Gdańsk, Poland; 3 Faculty of Chemistry, Adam Mickiewicz University in Poznań, Uniwersytetu Poznańskiego 8, 61-614 Poznań, Poland; paulina.debek@amu.edu.pl (P.S.); nowakiza@amu.edu.pl (I.N.); 4 Institute of Marine and Environmental Sciences, University of Szczecin, Mickiewicza 16A, 70-383 Szczecin, Poland; andrzej.witkowski@usz.edu.pl; 5 Centre for Modern Interdisciplinary Technologies, Nicolaus Copernicus University in Toruń, Wileńska 4, 87-100 Toruń, Poland

**Keywords:** diatom biosilica, palladium(II) nanoparticles, photocatalysis, methyl orange photodegradation

## Abstract

A new catalyst based on biosilica doped with palladium(II) chloride nanoparticles was prepared and tested for efficient degradation of methyl orange (MO) in water solution under UV light excitation. The obtained photocatalyst was characterized by X-ray diffraction, TEM and N_2_ adsorption/desorption isotherms. The photocatalytic degradation process was studied as a function of pH of the solution, temperature, UV irradiation time, and MO initial concentration. The possibilities of recycling and durability of the prepared photocatalysts were also tested. Products of photocatalytic degradation were identified by liquid chromatography–mass spectrometry analyses. The photocatalyst exhibited excellent photodegradation activity toward MO degradation under UV light irradiation. Rapid photocatalytic degradation was found to take place within one minute with an efficiency of 85% reaching over 98% after 75 min. The proposed mechanism of photodegradation is based on the assumption that both HO^•^ and O_2_^•−^ radicals, as strongly oxidizing species that can participate in the dye degradation reaction, are generated by the attacks of photons emitted from diatom biosilica (photonic scattering effect) under the influence of UV light excitation. The degradation efficiency significantly increases as the intensity of photons emitted from biosilica is enhanced by palladium(II) chloride nanoparticles immobilized on biosilica (synergetic photonic scattering effect).

## 1. Introduction

Every year, more than 0.7 million tons of dyestuffs and synthetic pigments are produced globally, among approximately 10,000 dyestuffs used on an industrial scale [1,2]. More than half of them are azo dyes (e.g., methyl orange), which are used by the textile and dye industry [3,4]. Therefore, in recent decades, elimination of azo dyes from wastewater has been a serious problem, as they contribute both to environmental pollution and to eutrophication, which affects the functioning of aquatic organisms [5,6,7]. The current methods used for wastewater treatment are mainly based on physicochemical techniques. These include chemical precipitation and subsequent separation of pollutants, electrocoagulation and adsorption. These techniques, however, do not ensure complete removal of the pollutants and may generate additional, secondary environmental pollution [8,9,10].

One of the most effective methods of removing organic compounds, including dyes, is the use of highly efficient and multi-stage technologies, which include advanced oxidation processes (AOPs) [11,12,13,14]. The mechanism of these processes is based on the use of high potentials of hydroxyl radicals HO^•^, which can be generated in various ways, e.g., [15]:

H_2_O_2_ + hv → 2HO^•^ (1)

H_2_O_2_ + O_2_ + hv → 2HO^•^ + O_2_^•−^ + H^+^ (2)

Fe(OH)^2+^ + hv → Fe^2+^ + HO^•^ (3)

One example of AOPs is photocatalysis. In this process, hydroxyl radicals are generated under the influence of UV radiation, the oxidizing effect of these radicals leads to degradation of many organic pollutants present in the environment. Photocatalytic recuperation takes place by converting photon energy into chemical energy [15,16,17].

In recent years, the degradation of toxic organic pollutants using heterogeneous photocatalysts has been intensively studied [18,19,20,21,22]. TiO_2_ has been considered one of the best photocatalysts [23]. However, its range of light response and photoelectric efficiency is limited due to the width of the energy gap—3.2 eV [23]. Apart from TiO_2_, other photocatalysts have been used in the process of degradation of organic pollutants and water splitting under the influence of visible light irradiation, including: BiVO_4_ [24], CdS [25], Bi_2_WO_6_ [26], gamma-C_3_N_4_ [27,28,29], BiFeO_3_ [30]. However, low specific surface area and fast rate of recombination of photogenerated charge carriers significantly reduce photocatalytic efficiency of these materials [17]. Therefore, one of the ways to increase the efficiency of a photocatalyst is to immobilize it on different media. Examples of such media are: glass or polymer fibres, silica, steel or glass plates, optical fibres or membranes. The material subjected to immobilization should meet the following criteria: it should be transparent to UV radiation, chemically resistant and easily separable from the solution [31].

The aim of this study was to develop an effective method of azo dye degradation, i.e., methyl orange (MO), based on a photocatalytic process using palladium(II) chloride-doped diatom biosilica and UV irradiation. The specific objectives of the study were: characterization of the kinetics of temperature-dependent photocatalytic degradation of MO, assessment of the efficiency of MO photocatalytic degradation depending on the pH of the solution and on the initial concentration of MO in the solution, identification of the products of photocatalytic degradation of MO, checking the possibility of recycling and evaluation of the durability of the prepared photocatalyst.

Diatoms are unicellular photosynthetic algae having shells (exoskeletons, frustules, carapaces) composed of amorphous, hydrated silica. These frustules consist of two overlapping shells called thecae (upper part—epitheca, lower—hypotheca) with a characteristic bilateral Petri dish-like structure [32,33,34,35,36].

The siliceous walls of diatom frustules are decorated by original pattern of ordered structural features such as pores, ridges, ribs, spikes or spines creating the most spectacular example of three-dimensional (3D) structured silica materials of biological origin. The diatom’s frustule surface contains a high level of the silanol groups (Si-OH) and siloxane bridges (Si-O-Si) [34,35,37].

The perfectly ordered three-dimensional structure, thermal and mechanical resistance, unique optical properties and biocompatibility make diatom biosilica a valuable raw material for the development of modern technologies, it can be used to produce optoelectronic devices, biosensors, gas sensors, catalysts, light harvesting materials, adsorbents, efficient filters, semiconductors, solar cells, templates for nanolithography, drug carriers or building materials in the synthesis of bone implants [33,36]. In addition, the multitude of applications of diatom biosilica may be due to the possibility of modifying its physicochemical properties to obtain new silica functional materials while maintaining a unique 3D structure [38]. Moreover, silica frustules isolated from cultures of diatoms show high photoluminescence activity associated with the emission in the mid-ultraviolet (290–300 nm) under UV irradiation, emission (493 nm) and excitation (480 nm) in the narrow blue region, and emission in the green region (498–525 nm) of the visible spectrum under UV irradiation [39].

In turn, catalysts modified with palladium compounds are often used in various organic reactions. The synthesis of palladium nanoparticles of various sizes (from 1 to tens of nanometres) and shapes, is carried out by chemical reduction [40,41] or electrochemical deposition [42] in aqueous solutions.

To the best of our knowledge, there are only a small number of literature reports describing the properties of palladium(II) nanoparticles made from chloride source. These compounds exhibit high thermal and mechanical stability and catalytic activity [43,44].

According to previously published reports, diatomaceous biosilica was functionalized with TiO_2_ to use as effective photocatalysts for indoor air purification [45,46], for photocatalytic degradation of rhodamine B [47,48], as catalyst for dye methyl blue photodegradation [49], and as photocatalysts in the abatement of acetaldehyde [50]. TiO_2_-doped diatom frustules has been tested for their activity in the photocatalytic degradation of the Congo red dye in water [51]. The produced diatom-FeOx composite was evaluated as catalyst in photodegradation of Rhodamine-6G [52]. Diatom biosilica modified with bio-inspired polydopamine and silver nanoparticles was used as active catalyst for dye degradation both, the cationic methylene blue and anionic Congo red dyes [53].

## 2. Results

### 2.1. Photocatalyst Characterization

**Figure 1:**
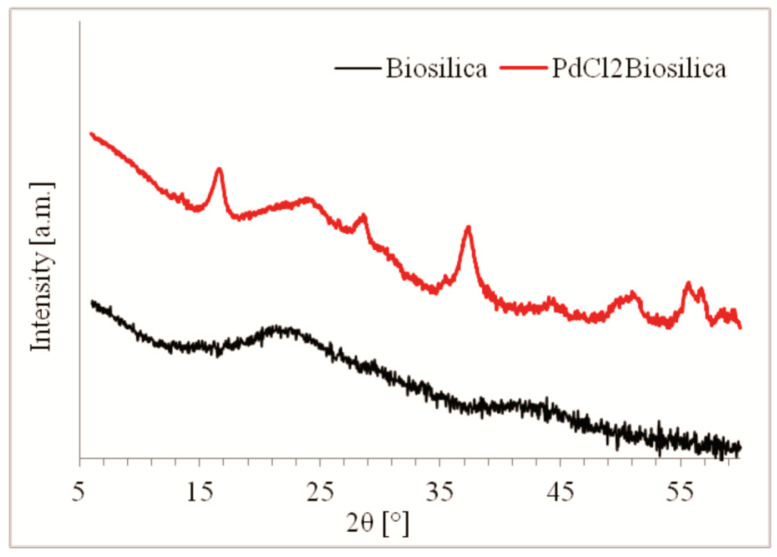
XRD patterns of the samples of pure diatom biosilica and palladium(II) chloride-doped diatom biosilica photocatalyst.

Figure 1 shows the X-ray diffraction patterns of diatom biosilica and palladium(II) chloride-doped diatom biosilica. The wide-angle diffractogram of pristine (unmodified material) shows one main reflection at 2θ = 22.3° indicating the amorphous nature of the biosilica. According to Sprynskyy at al. [39], this XRD pattern is characteristic for opal-A (biogenic amorphous silica).

The X-ray diffraction pattern of the palladium(II)-doped diatom biosilica, recorded in a wide-angle range of 2Θ permitted identification of palladium(II) chloride nanoparticles on the basis of the reflexes characteristic of this compound at 16.7°, 27.1°, 28.7°, 37.7°, 50.1°, 51.6°, 56.0°, 57.1° (Ref. Code: 00-001-0228).

**Figure 2:**
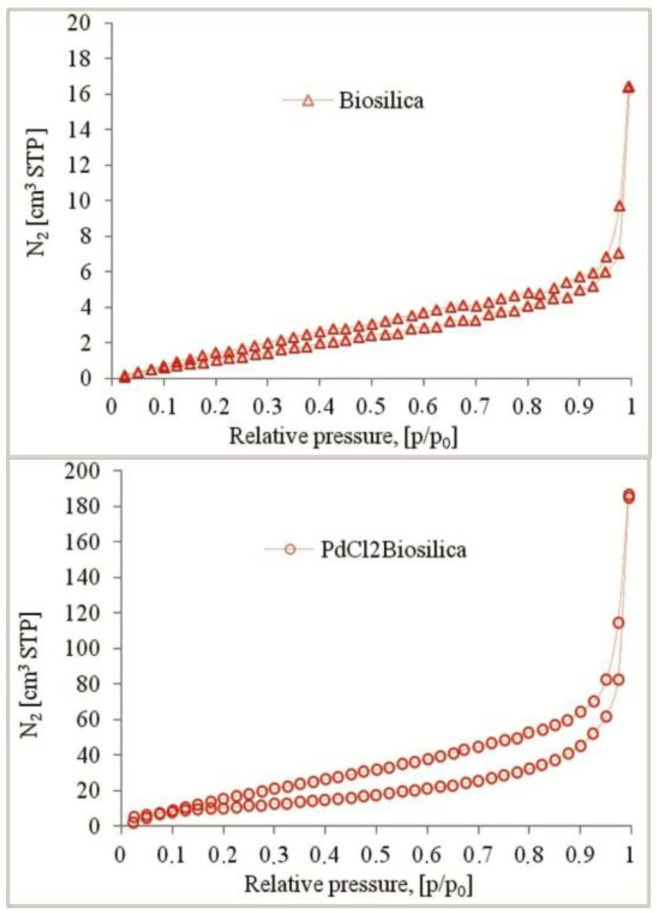
N_2_ adsorption isotherms of the pure diatom biosilica and palladium(II) chloride-doped diatom biosilica.

The low-temperature nitrogen adsorption/desorption isotherms of diatom biosilica and PdCl2 biosilica are presented in Figure 2. According to the IUPAC classification, the adsorption/desorption isotherm belongs to combination types I and II [54]. Type I is characteristic of microporous materials having relatively small external surfaces, while type II corresponds to nonporous or macroporous materials. The isotherm recorded for the obtained materials shows a hysteresis loop of H4 type, which indicates the presence of straight slit pores [55].

Table 1 shows the values of textural parameters such as the specific surface area, pore volume and average pore diameter obtained on the basis of the isotherm data. The specific surface area of the materials was determined using the BET (Brunauer–Emmett–Teller) method. Pore size distributions were calculated using the KJS (Kruk–Jaroniec–Sayari) method [56] based on the BJH (Barrett–Joyner–Halenda) algorithm.

The materials obtained have relatively low surface areas (BET), from ~30 m^2^/g for pure diatom biosilica to ~40 m^2^/g for palladium(II)-doped diatom biosilica. The average pore diameter varies from 3.93 to 4.45 nm. The total pore volume reaches over 0.28 cm^3^/g.

**Figure 3:**
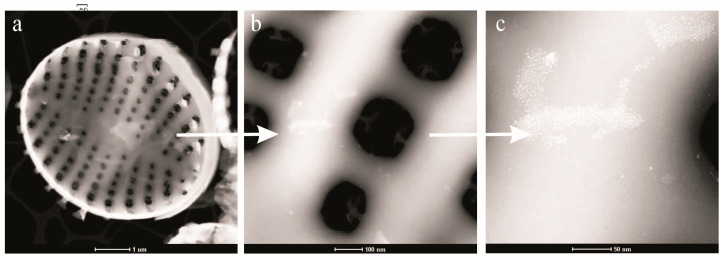
TEM micrographs of the palladium(II) chloride-doped diatom biosilica photocatalyst. Ordered periodic pore system (**a**), the morphology and structure of periodic pores (**b**), and the size, forms and specificity of the distribution of immobilized palladium(II) chloride nanoparticles (**c**).

The TEM micrographs (Figure 3) revealed the morphology and structure of the tested palladium(II) chloride-doped diatom biosilica photocatalyst and the palladium(II) chloride nanoparticles distribution.

The TEM images demonstrate the three-dimensional structure of a single diatom valve whose walls are perforated by an ordered periodic pore system (Figure 3a), the morphology and structure of periodic pores (Figure 3b), and the size, forms and specificity of the distribution of immobilized palladium(II) chloride nanoparticles (Figure 3c). The periodic pores are oval in shape, and their size ranges from 150 to 200 nm (arrow in Figure 3b). The arrow in Figure 3c shows that nanoparticles of palladium(II) chloride on the surface of diatom biosilica form single-layer clusters in the form of islets (flakes) of various configurations. These islets have dimensions ranging from about 10 to 100 nanometres along the long axis and contain from several to several hundred nanoparticles. The palladium(II) chloride nanoparticles with irregular shapes with diameters ranging of 2–3 nm adhere tightly to the biosilica surface. The very small size of the nanoparticles and their good dispersion on the biosilica surface can also contribute to the high efficiency of photodegradation.

### 2.2. Photocatalytic Activity

#### 2.2.1. MO Photocatalytic Degradation Depending on the pH Value

**Figure 4:**
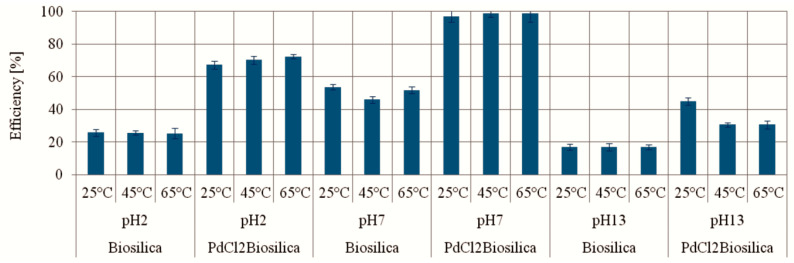
The efficiency of MO photocatalytic degradation for different pH value and temperature. Experimental conditions: t = 90 min; m (Biosilica)—10 mg; C_0_(MO)—9.98 mg/L; V (MO solution)–15 mL.

The dependence of MO photocatalytic degradation on pH values and temperature is shown in Figure 4. The photocatalytic activities of the PdCl2 biosilica and biosilica toward methyl orange was determined under UV light irradiation. Preliminary tests of the adsorption capacities of both the PdCl2 biosilica and biosilica in the dark showed a negligible effectiveness of a few percent. Similarly, it was found that the MO degradation does not occur in the dark and in the presence of TiO_2_ photocatalyst [57]. Initially blank test performed under UV irradiation without catalyst also showed negligible degradation effects.

**Figure 5:**
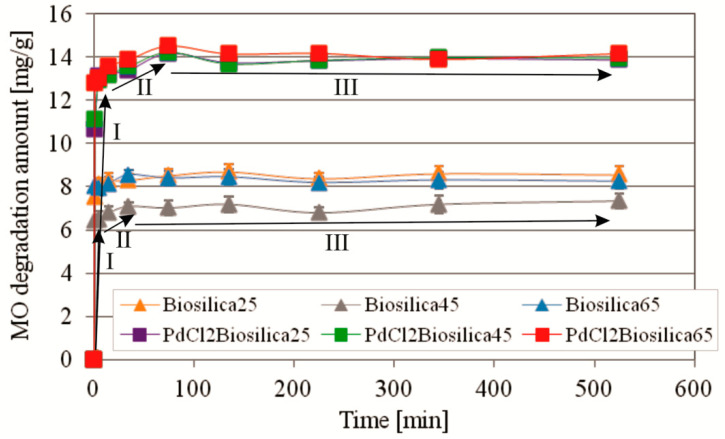
Kinetic curves of MO photocatalytic degradation under UV irradiation using PdCl2 biosilica and biosilica photocatalyst. Experimental conditions: m (Biosilica)—10 mg; T = 25 °C; C_0_(MO)—9.98 mg/L; V (MO solution)—15 mL; pH—7.0.

The results show that the biosilica and PdCl2 biosilica exhibit different abilities to induce photocatalytic degradation of MO in various pH conditions: acid, neutral and alkaline. The efficiency of MO degradation was almost twice higher for PdCl2 biosilica compared to unmodified biosilica under all pH conditions. The maximum efficiency of MO degradation (52% for biosilica and 98% for PdCl2Biosilica) was achieved in a neutral medium at pH 7. A fairly high efficiency (72%) of MO degradation in an acid medium was obtained in the presence of PdCl2Biosilica. The temperature increase in the range from 25 to 65 °C did not show a visible impact on the effectiveness of the photocatalytic degradation of the dye (see Figure 5).

Literature reports on methyl orange degradation in aqueous solutions using different types of photocatalysts give different pH values at which the maximum efficiency of the dye decomposition is achieved. The highest efficiency of dye degradation/sorption was obtained in an acidic medium at pH 3 using such photocatalysts as TiO_2_ [57,58], MnO_2_-decorated diatomite [59], polymeric chitosan-isovanillin [60], and at pH 2 using TiO_2_, Au/TiO_2_ composites [61] or Black Sand [62]. The optimum pH value for MO removal from aqueous solution using chitosan/diatomite composite was 5 [63]. Photodegradation of MO by chitosan embedded with nano-CdS was the most efficient (99%) at pH 4, but a high level of degradation (80%) was also observed at pH 6 and pH 8 [64]. The high degradation efficiency of MO in both strong acidic and near neutral environments was observed for Fe-based metallic glass [65]. Experimental results of MO photocatalytic degradation in the presence of TiO_2_/ASS also showed that the process rate was the highest at pH 7 [66]. The maximum efficiency of MO photodegradation was obtained for pH from the basic range, pH8 in the presence of TiO_2_ catalyst and at pH9 [67] in the presence of MnWO_4_ under UV radiation [68]. The increase in the conversion of MO with pH increasing from 6 to 11 was observed for MO photocatalytic degradation over TiO_2_ nanotubes [69]. The proposed explanation of this phenomenon is that the hydroxyl radicals formed in alkaline medium in the reaction (OH^−^ + h^+^ = OH^•^) can be more reactive toward the dye at higher pH [69]. However, according to the results obtained by Tokode and co-workers [70], the photonic efficiency of the MO degradation in the aqueous phase at the presence of TiO_2_ catalysts is comparable with that achieved in our study, irrespective of pH. This indicates that MO may be decomposed by both oxidative and reductive mechanisms in the photocatalytic process [70].

We hypothesise that both HO^•^ and O_2_^•−^ radicals as highly oxidizing species can be generated from diatom biosilica by attacks of emitted photons under UV light excitation and both of them can be involved in the reaction mechanism of MO degradation. The energy of the emitted photons can be absorbed by water molecules on the biosilica surface leading to generation of HO^•^ and O_2_^•−^ radicals. The emitted photons may also directly interact with the dye molecule, causing its degradation. In the process of MO degradation in the presence of diatom biosilica doped with PdCl_2_ nanoparticles, the photocatalytic effect was over twice as high as that in the presence of pure diatom basilica (Figure 4). As reported in [71], the photocatalytic activity of Bi_2_WO_6/_SiO_2_ photonic crystal film was observed to be about three times higher than that of the ordinary Bi_2_WO_6_ film in the photocatalytic decomposition of Rhodamine B (RhB) and phenol. These results confirmed a significant role of photonic crystals in photocatalytic processes. The photosensitive photonic crystals are promising for design and production of the synergistic photonic crystals based on plasmonic photocatalysts of plasmonic and photonic nanostructures [72].

#### 2.2.2. Kinetics of MO Photocatalytic Degradation at Different Temperatures

The kinetics of MO photocatalytic degradation at different temperatures under UV irradiation using PdCl2 biosilica and biosilica are shown in Figure 5. The kinetics is expressed by kinetic curves resealing three different stages of MO photocatalytic degradation. The initial rapid stage is followed by the second much slower process, and then by the stage of equilibrium with the weak fluctuation effects. The character of the process is very similar for biosilica and PdCl2 biosilica (the shape of the kinetic curves is the same), but the dye degradation efficiency is almost twice as high for PdCl2 biosilica as for Biosilica. In the initial step, rapid photocatalytic degradation of the test dye takes place within one minute with an efficiency of about 45% and 85% for biosilica and PdCl2Biosilica, respectively. The second stage lasts for about 35 min with the use of biosilica and about 75 min using PdCl2 biosilica. After reaching equilibrium, the maximum efficiency for the two tested basilica samples was 56% and 97%, for pristine and modified samples respectively. The high efficiency of MO photocatalytic degradation in the very rapid first stage may indicate that the surface layer reactions control the process. The contact time required to achieve equilibrium (75 min) was used in the next batch study of the isotherm of MO photocatalytic degradation over PdCl2 biosilica at 25 °C.

Similarly, high efficiency (95–99%) of MO photocatalytic degradation was achieved using Pt modified with TiO_2_ [73], Ag/HSTiO_2_ [74], chitosan with immobilized nano-CdS [64], TiO_2_-CuZSM-5 [75], scoria-Ni/TiO_2_ [76], and TiO_2_/ZnO mixture [77] after 90, 90, 80, 70, 45, 30 min UV radiation time, respectively. Significantly more time (240 min) was required to reach the maximum efficiency (90%) of MO degradation in the presence of graphene oxide supported titanium dioxide (GO/TiO_2_) composites [78]. A photocatalytic degradation rate of 87% in the same time of 240 min was observed for Cu-doped ZnO nanoparticles [79], while the highest photocatalytic efficiency (50%) of MnWO_4_ was obtained only after 480 min [68].

**Figure 6:**
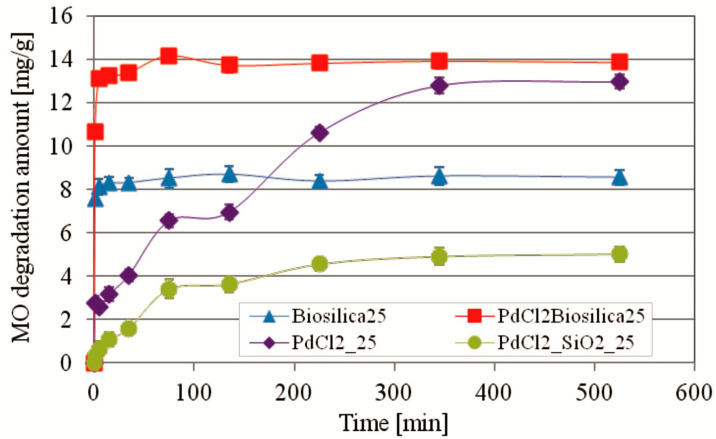
The comparison of the kinetics curves of MO photocatalytic degradation using PdCl2 biosilica and biosilica photocatalysts with the kinetics curves of MO photocatalytic degradation by pure PdCl2 and commercial amorphous silica doped with PdCl2. Experimental conditions: m (biosilica)—10 mg; T = 25 °C; C_0_(MO)—10.25 mg/L; V (MO solution)—15 mL; pH–7.0.

For comparison, the photocatalytic degradation of MO using only a palladium(II) chloride and commercial amorphous silica doped with PdCl2 (PdCl2_SiO2) as catalysts was also studied (Figure 6). The shape of the MO degradation kinetic curves using pure PdCl2 and biosilica doped with PdCl2 differs significantly. In the case of PdCl2 a much slower dye degradation process and a correspondingly much delayed time (345 min) to reach equilibrium were observed. Once equilibrium was reached, the maximum MO degradation efficiency over PdCl2 was 87%. Photocatalytic degradation of the dye by commercial amorphous silica also occurs much slower compared to pure biosilica or PdCl2 biosilica and the maximum efficiency of MO degradation during equilibrium is only 32%.

To explain the possible mechanisms of MO photocatalytic degradation in the presence of diatom biosilica doped with palladium(II) chloride nanoparticles, the experimental kinetics data were approximated with the pseudo-zero-order, Lagergren pseudo-first-order, pseudo-second-order, and Weber–Morris intraparticle diffusion models. According to determination coefficient values (R^2^) of the models fitting, the MO photocatalytic degradation was significantly better described by the pseudo-second-order kinetic model than by the pseudo-first-order kinetic model. This means that the degradation rate of the process is heterogenic and depends on several factors. The results of the study of photocatalytic degradation of MO in the presence of chitosan with immobilized nano-CdS [64], TiO_2_ nanoparticles [80], and polymeric chitosan-isovanillin [60] indicated that the kinetics of the photocatalytic degradation over these catalysts followed the pseudo-first-order kinetic model. The pseudo-zero-order kinetic model describes the first and second stages of the kinetic curves as linear functions and permits direct determination of values mg/g/min of photocatalytic degradation rate. The photocatalytic degradation rate in the first stage is almost hundred times higher than that in the second stage. According to the obtained results of Weber–Morris model fitting, two mechanisms are involved in the kinetic processes of photocatalytic degradation. The value of constant A indicates that the majority of MO is retained in the boundary layer and suggest that the photocatalytic degradation rate is mainly governed by the external surface reactions. This mechanism dominates in the kinetics of MO photocatalytic degradation. The intra-particle diffusion (corresponding to stage II on the kinetic curves) in the porous biosilica is insignificant.

#### 2.2.3. MO Photocatalytic Degradation Depending on the MO Initial Concentration

**Figure 7:**
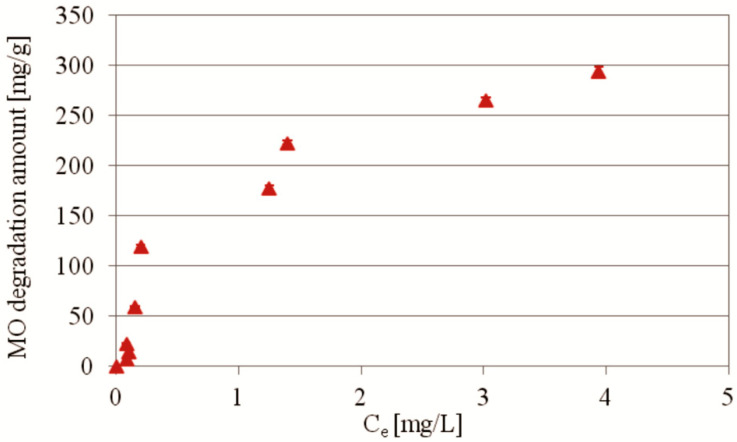
Isotherm of MO photocatalytic degradation as a function of the initial concentration of MO in the presence of PdCl2 biosilica photocatalyst. Experimental conditions: m (biosilica)—10 mg; t = 75 min; T = 25 °C; C_0_(MO)—5–200 mg/L; V (MO solution)—15 mL; pH—7.0.

**Figure 8:**
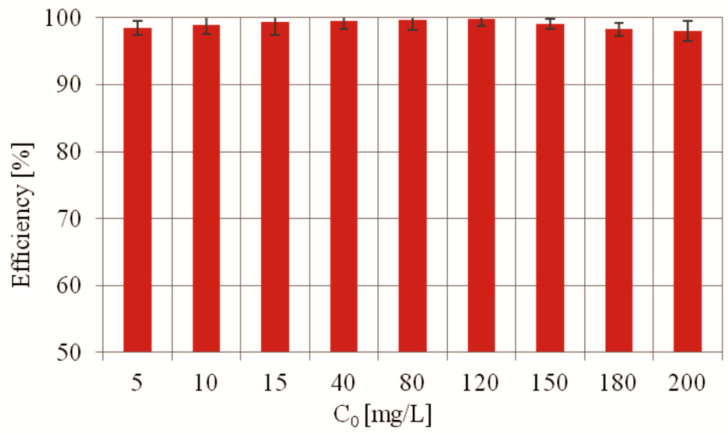
Efficiency of MO photocatalytic degradation as a function of the initial concentration of MO in the presence of PdCl2 biosilica photocatalyst. Experimental conditions: m (biosilica)—10 mg; T = 25 °C; t = 75 min; C_0_(MO)—5–200 mg/L; V (MO solution)—15 mL; pH—7.0.

The isotherm and efficiency of MO photocatalytic degradation under UV irradiation at 25 °C in the presence of PdCl2 biosilica as a function of dye initial concentration are shown in Figure 7 and Figure 8, respectively.

The isotherm of MO degradation in the presence of PdCl2 biosilica photocatalyst was determined for the initial concentrations 5–200 mg/L. The results indicate that the amount of converted dye rises steeply with increasing dye initial concentration (Figure 7). Interestingly, the steep character of the slope of the isotherm of the photocatalytic degradation of MO remains even in the presence of the highest concentrations of the dye used in the experiment, which may indicate a sufficient energy potential of this photocatalyst for effective decomposition of even higher dye concentrations. The maximum conversion of MO of 294 mg/g was reached in this experiment at 200 mg/L of the dye initial concentration. Three isotherm models (Langmuir, Freundlich, and Temkin) were applied to describe the experimental data. The results of the isotherm models fitting (Table 2) show that the MO degradation by PdCl2 biosilica photocatalyst could be best described by the Temkin model with high determination coefficients. The fits to the Freundlich and Langmuir models were of poor quality, according to the evaluated fitting constants. According to the Temkin isotherm theory, we can assume similar values of energy of photocatalytic degradation process for the whole range of the used initial concentrations and the results indicate that this process is irreversible.

The efficiency of MO degradation in the presence of PdCl2 biosilica photocatalyst was determined to be at the level of 98–99.8% for the initial concentration ranges 5–200 mg/L (Figure 8). The results show that the degradation efficiency does not decrease appreciably with increasing MO initial concentration up to 200 mg/L. This phenomenon can be explained by the fact that the photocatalytic capacity of the synthesized catalyst is very high, and the dye degradation products do not interfere with the photocatalytic degradation process.

According to the published reports, high effectiveness (80–99%) of MO photocatalytic degradation can be achieved also by employing other photocatalysts. The degradation efficiency 98% of MO photocatalytic degradation was reached using Fe_3_O_4_ nanoparticles [81], Pt modified TiO_2_ [74], and TiO_2_-CuZSM-5 mixture [76], 99% by Ag/HSTiO_2_ catalyst [70], and chitosan with immobilized nano-CdS photocatalyst [44], 96% by scoria-Ni/TiO_2_ [76], 93% by employing SiO_2_-TiO_2_ photocatalyst [82], and 87% using Cu-doped ZnO nanoparticles [79]. Significantly lower efficiency (50–70%) of MO degradation was found when using such photocatalysts as Ag/TiO_2_ nanocomposites [83], anatase TiO_2_ nanoparticles [80], MnWO_4_ powder [68], and ZnO/GO and TiO_2_/ZnO/GO nanocomposite photocatalyst [84]. However, in order to improve the quality of most of the tested catalysts, it is necessary to reduce the photocatalytic degradation time and increase the recycling time.

#### 2.2.4. The Recyclability of the Used PdCl2 Biosilica Photocatalyst

**Figure 9:**
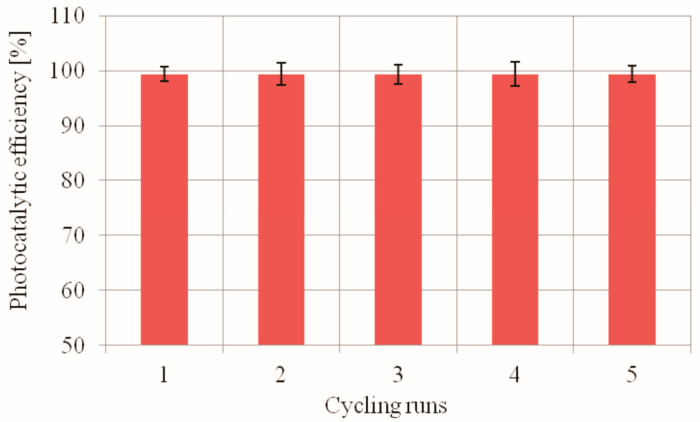
Efficiency of MO degradation as a function of UV irradiation time over PdCl2 biosilica photocatalyst. Experimental conditions: m (biosilica)—10 mg; C_0_(MO)—9.98 mg/L; T = 25 °C; t = 75 min; V (MO solution)—15 mL; pH—7.0.

The stability of photocatalyst recycling capacity is an important indicator of its possible application. Five cycling runs of the photocatalytic degradation of MO dye was performed to evaluate the photocatalytic stability of reused photocatalyst. The results concerning the reusability of PdCl2 biosilica photocatalyst are shown in Figure 9. As indicated, a high MO photodegradation is maintained after five cycle rounds of the photocatalyst reuse, which proved that the prepared PdCl2 biosilica photocatalyst has a very good recyclability. The fears that immobilized nanoparticles of palladium(II) chlorides may be insufficiently stable in an aqueous solution of the dye were not confirmed in our experiment.

Palladium(II) chloride anchored on polystyrene modified with 5-amino-1,10-phenanthroline (PS-phen/PdCl_2_) was used as an efficient recoverable catalyst for Suzuki cross-coupling reactions and after the catalyst reuse in five cycles no significant Pd leaching and no loss of its catalytic activity were observed [43]. Moreover, a comparison of the catalytic performance of PS-phen/PdCl_2_ and other supported Pd catalysts indicated that PS-phen/PdCl_2_ was more active and more stable than the commonly used supported catalyst Pd/C [43]. This observation was explained by probable formation of covalent interactions between PdCl_2_ and the nitrogen-containing ligand of the support [43]. A similar catalytic efficiency of Pd particles and PdCl_2_ in the reduction of methylene blue by hydrazine has been reported by Aoki and co-authors [44].

### 2.3. Methyl Orange Degradation Products

**Figure 10:**
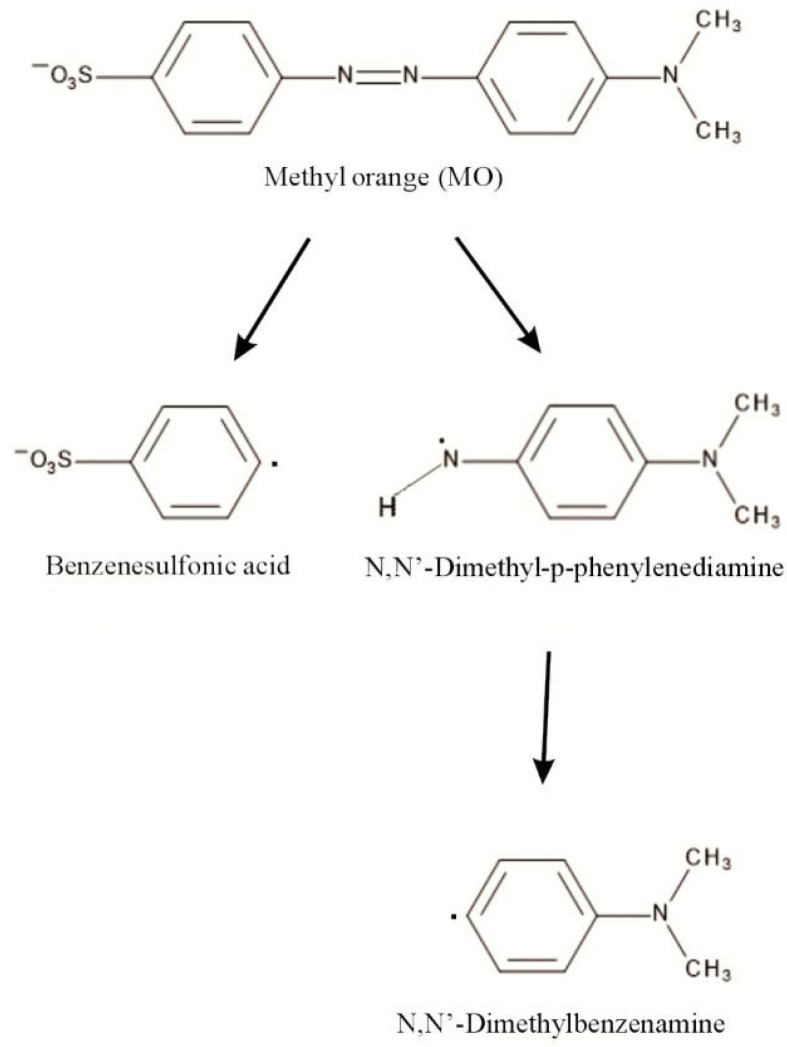
Degradation scheme of methyl orange (MO).

Figure 10 presents the scheme of degradation of methyl orange (MO) obtained on the basis of the results of ESI-HRMS.

Electrospray Ionization–High-Resolution Mass Spectrometry (ESI-HRMS) for the standards of: methyl orange, benzenesulphonic acid, *N*,*N*-dimethyl-p-phenylenediamine and *N*,*N*-dimethylbenzenamine and MO reaction mixture was carried out in ESI(+), ESI(+) + FA (formic acid) and ESI(−) modes.

Methyl orange in ESI(−) mode gives a peak at a *m*/*z* value of 304.0760, with the molecular formula of C_14_H_14_N_3_SO_3_ (M_MO_, Figure S1) and in ESI(+) or ESI(+) +FA it corresponds to *m*/*z* value of 306.0918 of the molecular formula of C_14_H_16_N_3_SO_3_ (M_MO_H_2_, Figures S6 and S11). A similar peak was detected at an *m*/*z* value of 304 for MO standard by Baiochhi et al. [85] using MS/MS and MS/MS/MS analyses in ESI(−) mode and at *m*/*z* ratio of 304.2 by Chen et al. [86] using MS/MS. The ion at *m*/*z* 156.9978 (M_BA_, benzenesulphonic acid radical anion, ESI(−), Figure S2) corresponds to [C_6_H_5_O_3_S] and the ion at *m*/*z* 159.0107 (M_BA_H_2_) corresponds to C_6_H_5_O_3_SH_2_ (M_BA_H_2_, benzenesulphonic cation radical, ESI(+) or ESI(+) + FA, Figures S7 and S12). *N*,*N*-dimethyl-p-phenylenediamine protonated standard with *m*/*z* value of 137.1072 corresponding to C_8_H_11_N_2_H (M_D_H), of the molecular formula of C_8_H_13_N_2_ is detected in ESI(+) and ESI(+) + FA (Figure S8 and S13). The *N*,*N*-dimethyl-p-phenylenediamine radical anion standard of the molecular formula of C_8_H_11_N_2_H (M_D_, *m*/*z* = 135.0882, ESI(−)) is shown in Figure S3.

The primary peak assigned to *N*,*N*-dimethylbenzenamine anion radical standard is detected in ESI(−) mode with *m*/*z* value of 120.0439 corresponding to C_8_H_10_N (M_DB_, Figure S4). In Figures S9 and S14, the ion at *m*/*z* = 122.0969 (ESI(+) + (ESI(+) +FA), corresponding to *N*,*N*-dimethylbenzenamine radical cation standard of the molecular formula C_8_H_10_NH_2_ (M_DB_H_2_), is shown.

The mass spectrum for the full-range scan of MO reaction in ESI(−) mode is shown in Figure S5. As the obtained results imply, the first stage of the methyl orange degradation involves the azo bond breaking, which leads to the formation of benzenesulphonic acid radical anion (*m*/*z* value of 156.9950). In negative-ion mode, only this peak is detected.

Similarly, in ESI(+) and ESI(+) + FA, a peak corresponding to benzenesulphonic acid radical cation is detected, as shown in Figures S10 and S15 (*m*/*z* = 159.9701). The peak at an *m*/*z* value of 122.0969 (M_DB_H_2_), shown in Figures S10 and S15, corresponds to the radical cation of *N*,*N*-dimethylbenzenamine. *N*,*N*-dimethyl-p-phenylenediamine radical cation with *m*/*z* value of 137.1072 (M_D_H_2_) corresponding to C_8_H_13_N_2_H, with the molecular formula of C_8_H_13_N_2_ is detected, as shown in Figures S10 and S15 [87]. Summary of ESI-HRMS results is presented in Table 3.

## 3. Materials and Methods

### 3.1. Preparation of the Photocatalyst

#### 3.1.1. Diatom Biosilica

Diatom biosilica was obtained under laboratory conditions by cultivation of selected diatom species *Pseudostaurosira trainorii* (the Culture Collection of Baltic Algae, Institute of Oceanography, University of Gdańsk, Poland) according to the procedure described in the work of M. Sprynskyy and co-workers [39]. The diatom biosilica was fabricated in the form of single diatom frustules of elliptical shapes with an average valve length r 4–5 μm. The diatom silica has a three-dimensional structure with silica walls perforated by spatially periodic network of oval pores with pore size range 150–200 nm [39,88]. According to X-ray diffraction analysis data, this biosilica was identified as hydrated silica like opal-A [39]. The physicochemical properties of the obtained biosilica are characterized in more detail in previously published works [39,88].

#### 3.1.2. Palladium(II) Chloride-Doped Diatom Biosilica/Commercial Amorphous Silica

The synthesis of diatom biosilica doped with palladium(II) chloride was performed by dispersing the diatom biosilica or commercial amorphous silica (POCH) (0.5 g) using ultrasound bath for 3 h, in a mixture containing 75 mL of methanol (Honeywell) and palladium(II) chloride (1 wt.%; PdCl_2_, Alfa Aesar, 99.9%). Next, the mixture was subjected to stirring for 24 h at 25 °C and then the catalysts were dried at 60 ºC to evaporate the solvent.

### 3.2. Photocatalyst Characterization

The crystal structure of the samples was investigated using X-ray diffraction (Bruker AXS D8 Advance diffractometer) with Cu Kα (0.154 nm) radiation at a scan rate of 2θ = 4–60° with a step of 0.05°. The morphology and structure of the doped diatom biosilica, as well as palladium(II) chloride distribution in the synthesized catalysts, were examined by transmission electron microscopy (TEM, FEI Tecnai F20 X-Twin tool).

Low-temperature nitrogen adsorption/desorption isotherms were recorded on Quantachrome Autosorb iQ at −176 °C, after prior degassing of the sample in vacuum at 120 °C for 3 h. The surface area of the catalysts was determined by the BET (Brunauer–Emmet–Teller) method. The pore volume was determined using the KJS-BJH method based on the BJH (Barret–Joyner–Halenda) algorithm.

### 3.3. Photocatalytic Activity

The photocatalytic activity of palladium(II) chloride-doped biosilica (PdCl2Biosilica), palladium(II) chloride and commercial amorphous silica doped with palladium(II) chloride (PdCl2_SiO2), was evaluated in photocatalytic degradation of methyl orange (Sigma-Aldrich, 4-[4-(Dimethylamino)phenylazo]benzenesulfonic acid sodium salt, MO) in water solution (pH7), hydrochloric acid solution (0.2 M; pH2, Stanlab) or sodium hydroxide solution (0.1 M; pH13, Stanlab) under UV light irradiation generated by a 125 W Hg lamp with a 365 nm cutoff filter.

In each experiment, 0.01 g of the photocatalyst was mixed with 15 mL of methyl orange solution (with various concentrations of MO: 5, 10, 15, 40, 80, 120, 150, 180, 200 mg L^−^^1^). The process was conducted at three different temperatures: 25 °C, 45 °C and 65 °C.

At irradiation time intervals of 0, 1, 5, 15, 35, 75, 135, 225, 345 and 525 min, the suspensions were collected and filtered to remove the photocatalyst particles. The MO concentrations were monitored at 464 nm during the adsorption/photodegradation process using a UV–Vis spectrophotometer (Varian Cary 50).

#### The Stability of the Used Photocatalysts

The PdCl2 biosilica catalyst previously used in the process of MO degradation was subjected to regeneration. The process was carried out according to the description given in Section 2.3. Initially, the used catalyst was washed on a fine mesh size filter (to separate the catalyst from the MO solution). Next, the catalyst that remained on the filter was rinsed with ethanol and finally dried in a laboratory dryer. The regenerated catalyst was reused in the MO degradation process under identical conditions. The same catalyst was used 5 times, as this number of reuses was allowed by the amount of regenerated catalyst. The volume of MO solution used in the reaction was adjusted in proportion to the amount of the remaining catalyst.

### 3.4. Electrospray Ionization–High-Resolution Mass Spectrometry (ESI-HRMS)

To identify the photodegradation products of MO, Electrospray Ionization–High-Resolution Mass Spectrometry (ESI-HRMS) analyses were performed. After the photocatalysis process, the solution of MO was filtered through a 0.22 μm syringe filter (Chemland). The degradation products were identified according to the parameters described below. The measurements were carried out using a High-Resolution Triple TOF spectrometer (AB Sciex, 5600) in ESI negative, positive and positive with formic acid (HA) mode. Formic acid (1 μL/20 mL of sample) was added to facilitate ionization.

Each spectrum was scanned from 100 to 700 *m*/*z*.

## 4. Conclusions

The prepared diatom biosilica doped with palladium(II) chloride nanoparticles (PdCl2Biosilica) is capable of acting as a highly efficient photocatalyst of MO degradation in water solution under UV light excitation. The palladium(II) chloride nanoparticles of irregular shapes with diameters ranging from 2 to 3 nm adhered tightly to the biosilica surface in the form of single-layer clusters.

The degradation efficiency was determined for PdCl2 biosilica to be at a level of 98–99.8% for the MO initial concentration ranging from 5 to 200 mg/L with a maximum MO conversion of 294 mg/g. For comparison, the maximum MO degradation efficiency of 52% was detected for pure biosilica (biosilica). The degradation process was the most intense in a neutral medium at pH7. The recycling experiment showed the stability of the degradation efficiency for PdCl2 biosilica after five consecutive runs.

The kinetics curves of MO photocatalytic degradation over PdCl2 biosilica or biosilica can be divided into three different stages: initial rapid photocatalytic degradation, slowly running process, and the stage of equilibrium with weak fluctuation effects. The photocatalytic process was very fast and in the initial step the degradation efficiency reached about 45% for biosilica and 85% for PdCl2 biosilica within the first minute.

PdCl2 biosilica and biosilica showed negligible abilities to adsorb MO in the dark. The effect of UV radiation on the degradation of MO in the absence of a catalyst and the effect of temperature increase were also negligible. Therefore, it is reasonable to suppose that both HO^•^ and O_2_^•−^ radicals, highly oxidizing species, can be generated on the biosilica surface by attacks of photons emitted from pure diatom biosilica under UV light excitation and involved in the photocatalytic processes of MO degradation (photonic scattering effect). The photocatalytic activity of PdCl2 biosilica is effectively enhanced (over twice) compared to that of pure biosilica, which can be interpreted as a result of the photonic effect of palladium(II) chloride nanoparticles immobilized on biosilica (synergetic photonic scattering effect).

## Figures and Tables

**Table 1:** Porous structure parameters of the samples studied.

Sample	Specific Surface Area [m^2^ g^−1^]	Pore Volume [cm^3^ g^−1^]	Average Pore Diameter [nm]
Biosilica	30	0.43	3.93
PdCl2Biosilica	40	0.28	4.45

**Table 2:** The parameters of the MO photocatalytic degradation isotherm as determined from fitted isotherm models: C_e_ (mg/L) is the equilibrium concentration, q_m_ (mg/g) the maximum adsorption capacity, K_L_ (L/mg) the Langmuir constants, K_F_ (L/g) and 1/n are the Freundlich constants, BT is a constant related to the heat of adsorption and defined by the expression BT = RT/b, b is the Temkin constant (J/mol), A_T_ is the Temkin isotherm constant (L/g), T is the absolute temperature (K), and R is the gas constant (8.314 J/mol K).

Kinetics Model	Equation	Parameters	Value	R^2^
PdCl2Biosilica25				
Langmuir	C_e_/q_e_ = (1/q_m_K_L_) + C_e_/q_m_	K_L_ (L/mg) q_m_ (mg/g)	0.5442 434.8	0.5047
Freundlich	logq_e_ = logK_F_ + 1/n logC_e_	K_F_ (L/g) 1/n	137.27 0.767	0.7707
Temkin	q_e_ = B_T_lnA_T_ + B_T_lnC_e_	B_T_ (J/mol) A_T_ (L/g)	69.0 15.203	0.9634

**Table 3:** Summary of ESI-HRMS results.

*m*/*z*	Compound	ESI Mode	Molecular Formula	Symbolic Formula
304.0760	MO standard	(−)	C_14_H_14_N_3_SO_3_	M_MO_
306.0918	MO protonated standard fragmentation product	(+) and (+) + FA	C_14_H_16_N_3_SO_3_	M_MO_H_2_
156.9966	Benzenesulphonic acid radical anion standard	(−)	C_6_H_5_O_3_S	M_BA_
159.0107	Benzenesulphonic acid protonated standard	(+) and (+) + FA	C_6_H_5_O_3_SH_2_	M_BA_H_2_
135.0882	*N*,*N*-dimethyl-p-phenylenediamine radical anion standard	(−)	C_8_H_11_N_2_H	M_D_
137.1072	*N*,*N*-dimethyl-p-phenylenediamine radical cation standard	(+) and (+) + FA	C_8_H_11_N_2_H_2_	M_D_H
120.0439	*N*,*N*-dimethylbenzenamine anion radical standard	(−)	C_8_H_10_N	M_DB_
122.0969	*N*,*N*-dimethylbenzenamine cation radical standard	(+) and (+) + FA	C_8_H_10_NH_2_	M_DB_H_2_

## Data Availability

The data presented in this study are available upon request.

## Supplementary Materials

The following are available online at https://www.mdpi.com/article/10.3390/ijms22136734/s1, Figure S1: ESI-MS(-) of MO standard with molecular formula C14H14N3SO3 (MMO, *m*/*z* – 304.0760), Figure S2: ESI-MS(-) of benzenesulphonic acid radical anion standard with molecular formula C6H5O3S (MBA, *m*/*z* – 156.9978), Figure S3: ESI-MS(-) of N,N-dimethyl-p-phenylenediamine radical anion standard with molecular formula C8H11N2H (MD, *m*/*z*=135.0882), Figure S4: ESI-MS(-) of N,N-dimethylbenzenamine radical anion standard with molecular formula C8H10N (MDB, *m*/*z* = 120.0439), Figure S5: ESI-MS(-) of MO reaction mixture, Mass/Charge – 100-600 [Da], Figure S6: ESI-MS(+) of MO standard with molecular formula C14H16N3SO3 (MMOH2, *m*/*z*=306.0918), Figure S7: ESI-MS(+) of benzenesulphonic acid radical cation standard with molecular formula C6H5O3SH2 (MBAH2, *m*/*z* – 159.0107), Figure S8: ESI-MS(+) of N,N-dimethyl-p-phenylenediamine radical cation standard with molecular formula, C8H11N2H2 (MDH, *m*/*z* – 137.1072), Figure S9: ESI-MS(+) of N,N-dimethylbenzenamine radical cation standard with molecular formula C8H10NH2 (MDBH2, *m*/*z* = 122.0969), Figure S10: ESI-MS(+) of MO reaction mixture, Mass/Charge – 100-700 [Da], Figure S11: ESI-MS(+) + FA of MO standard with molecular formula C14H16N3SO3 (MMOH2, *m*/*z*=306.0918), Figure S12: ESI-MS(+) + FA of benzenesulphonic acid radical cation standard with molecular formula C6H5O3SH2 (MBAH2, *m*/*z* – 159.0107), Figure S13: ESI-MS(+) + FA of N,N-dimethyl-p-phenylenediamine radical cation standard with molecular formula, C8H11N2H2 (MDH, *m*/*z* – 137.1072), Figure S14: ESI-MS(+) + FA of N,N-dimethylbenzenamine rad-ical cation standard with molecular formula C8H10NH2 (MDBH2, *m*/*z* = 122.0969), Figure S15: ESI-MS(+) + FA of MO reaction mixture, Mass/Charge – 100-700 [Da].
