# Supplementary material for: Diatom Biosilica Doped with Palladium(II) Chloride Nanoparticles as New Efficient Photocatalysts for Methyl Orange Degradation
Source: Int J Mol Sci. 2021 Jun 23;22(13):6734. doi: 10.3390/ijms22136734 (PMC8267799; doi:10.3390/ijms22136734)
Supplement: Supplementary file 1 [file ijms-22-06734-s001.zip › ijms-1262198-supplementary.pdf]

SUPPLEMENTARY MATERIALS

Article

Diatom Biosilica Doped with Palladium(II) Chloride Nanoparticles as New Efficient Photocatalysts for Methyl Orange Degradation

Myroslav Sprynskyy [1,2,*], Paulina Szczyglewska [3], Izabela Wojtczak [1,2], Izabela Nowak [3], Andrzej Witkowski [4],
Bogusław Buszewski [1,5] and Agnieszka Feliczak-Guzik [3,*]

[1] Faculty of Chemistry, Nicolaus Copernicus University in Toruń, 7 Gagarina Str, 87-100 Toruń, Poland;
izabelawojtczak1991@gmail.com (I.W.); bbusz@chem.umk.pl (B.B.)
[2] Department of Biopharmaceutics and Pharmacodynamics, Medical University of Gdańsk, Hallera 107,
80-416 Gdańsk, Poland
[3] Faculty of Chemistry, Adam Mickiewicz University in Poznań, Uniwersytetu Poznańskiego 8,
61-614 Poznań, Poland; paulina.debek@amu.edu.pl (P.S.); nowakiza@amu.edu.pl (I.N.)
[4] Institute of Marine and Environmental Sciences, University of Szczecin, Mickiewicza 16A, 70-383 Szczecin, Poland; andrzej.witkowski@usz.edu.pl
[5] Centre for Modern Interdisciplinary Technologies, Nicolaus Copernicus University in Toruń, Wileńska 4, 87-100 Toruń, Poland
* Correspondence: mspryn@chem.umk.pl (M.S.); agaguzik@amu.edu.pl (A.F.-G.);
Tel.: +48-618-291-747 (A.F.-G.)

The mass spectra and compound confirmation for the degradation products of MO.

NEGATIVE – ION MODE

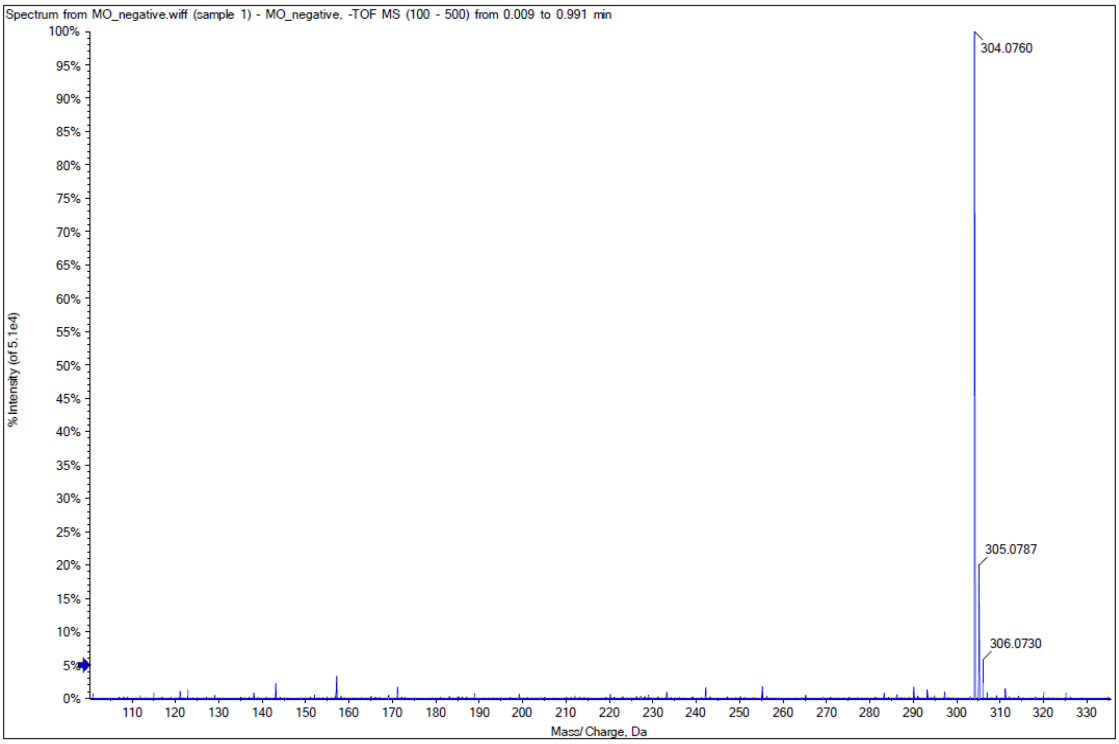

Figure S1. ESI-MS(-) of MO standard with molecular formula $C_{14}H_{14}N_3SO_3$ (M_{MO}, m/z – 304.0760)

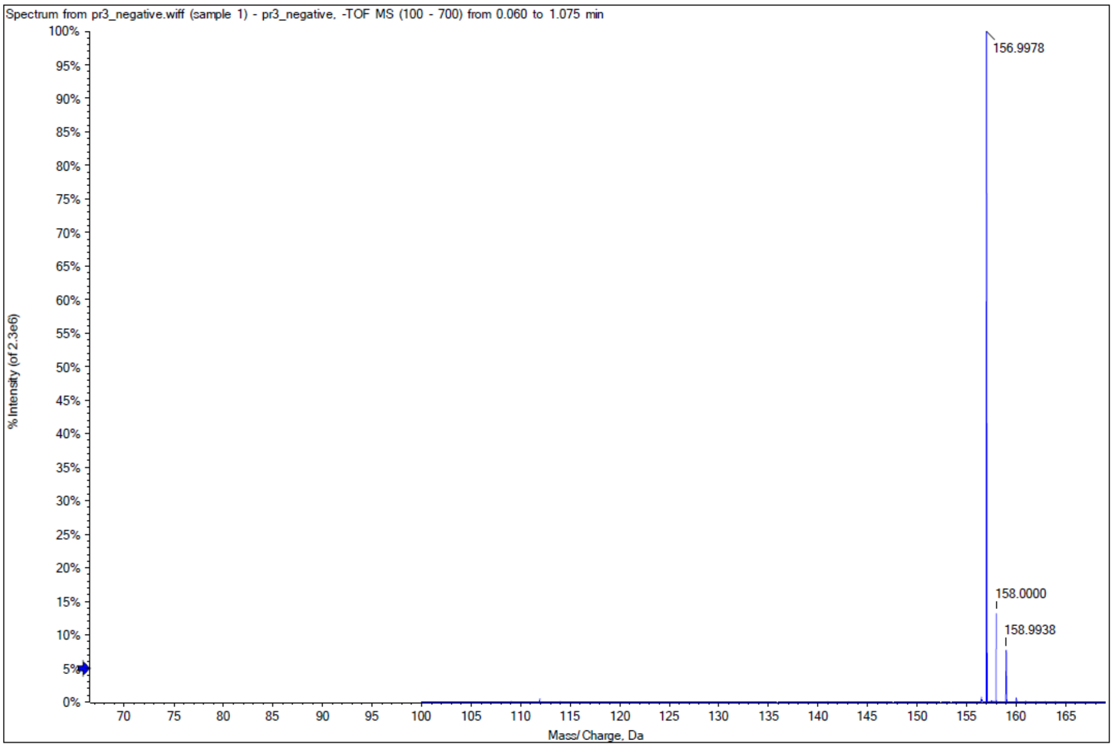

Figure S2. ESI-MS(-) of benzenesulphonic acid radical anion standard with molecular formula $C_6H_5O_3S$ (M_{BA}, m/z – 156.9978)

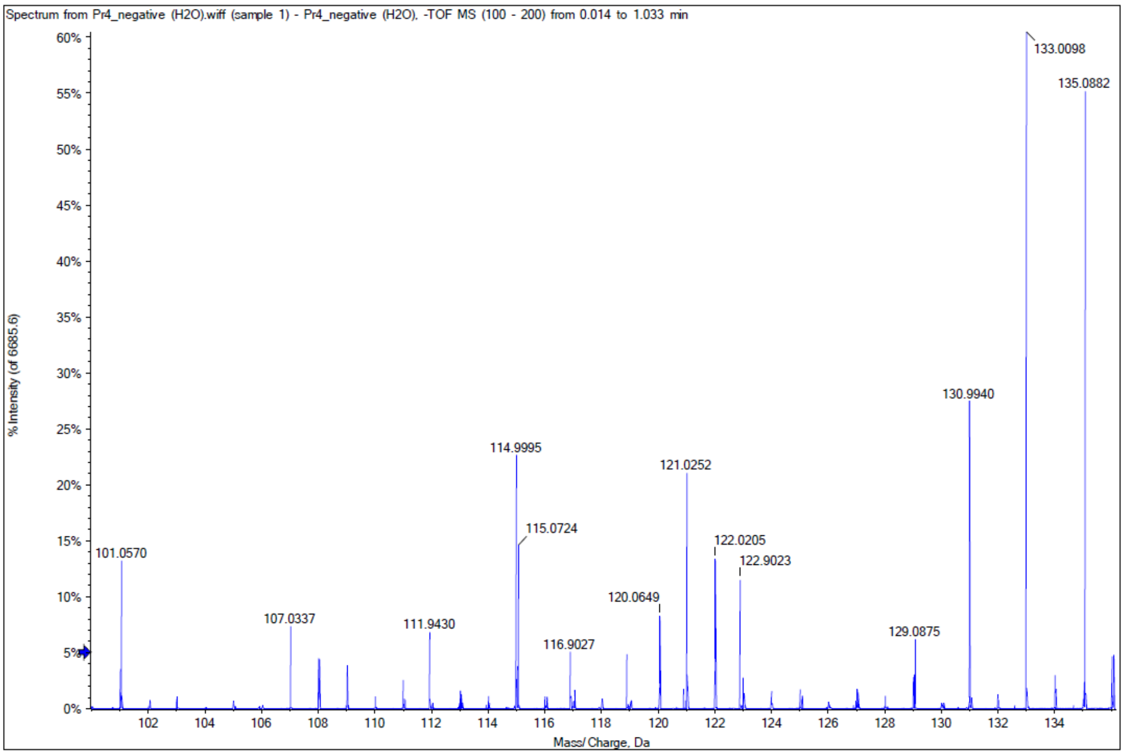

Figure S3. ESI-MS(-) of N,N-dimethyl-p-phenylenediamine radical anion standard with molecular formula $C_8H_{11}N_2H$ (M_D, m/z=135.0882)

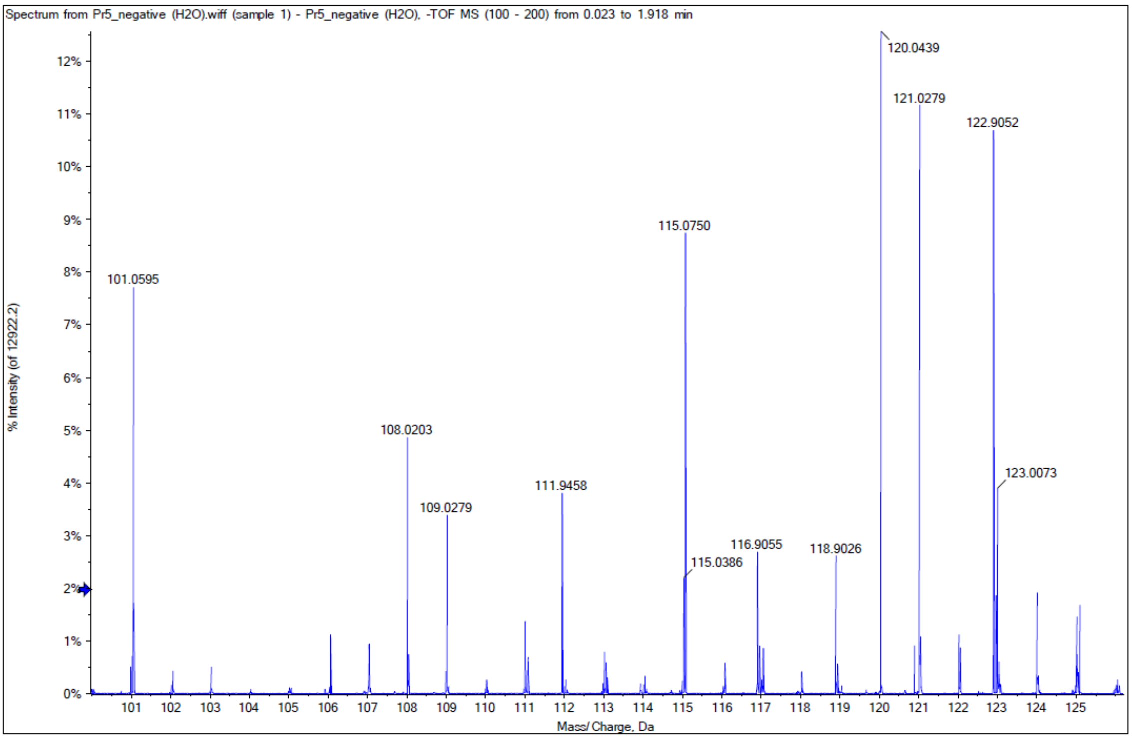

Figure S4. ESI-MS(-) of N,N-dimethylbenzenamine radical anion standard with molecular formula $C_8H_{10}N$ (M_{DB}, m/z = 120.0439)

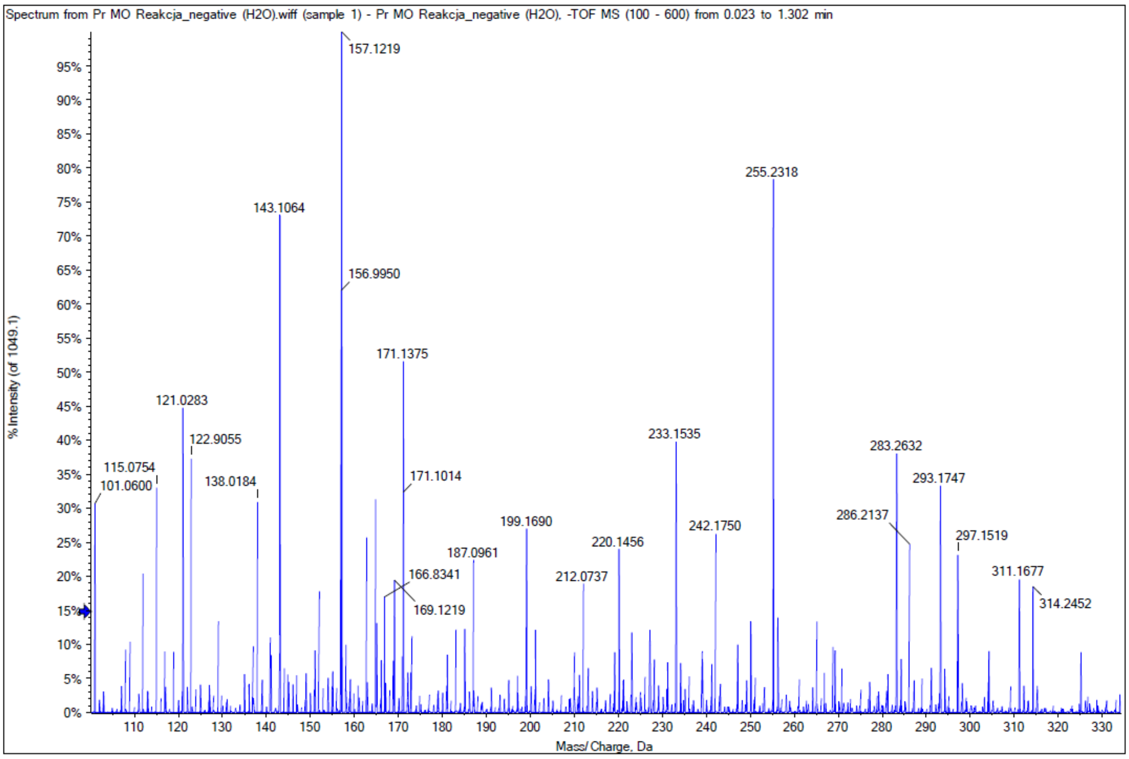

Figure S5. ESI-MS(-) of MO reaction mixture, Mass/Charge – 100-600 [Da]

POSITIVE– ION MODE

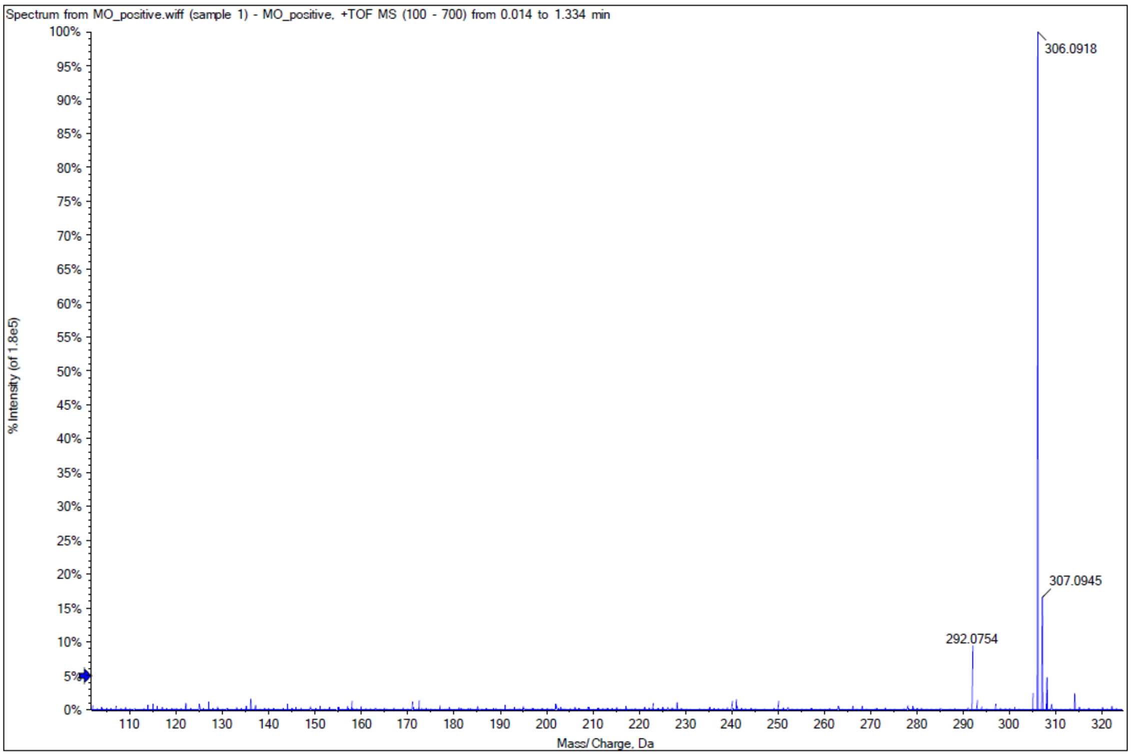

Figure S6. ESI-MS(+) of MO standard with molecular formula $C_{14}H_{16}N_3SO_3$ ($M_{MO}H_2$, m/z=306.0918)

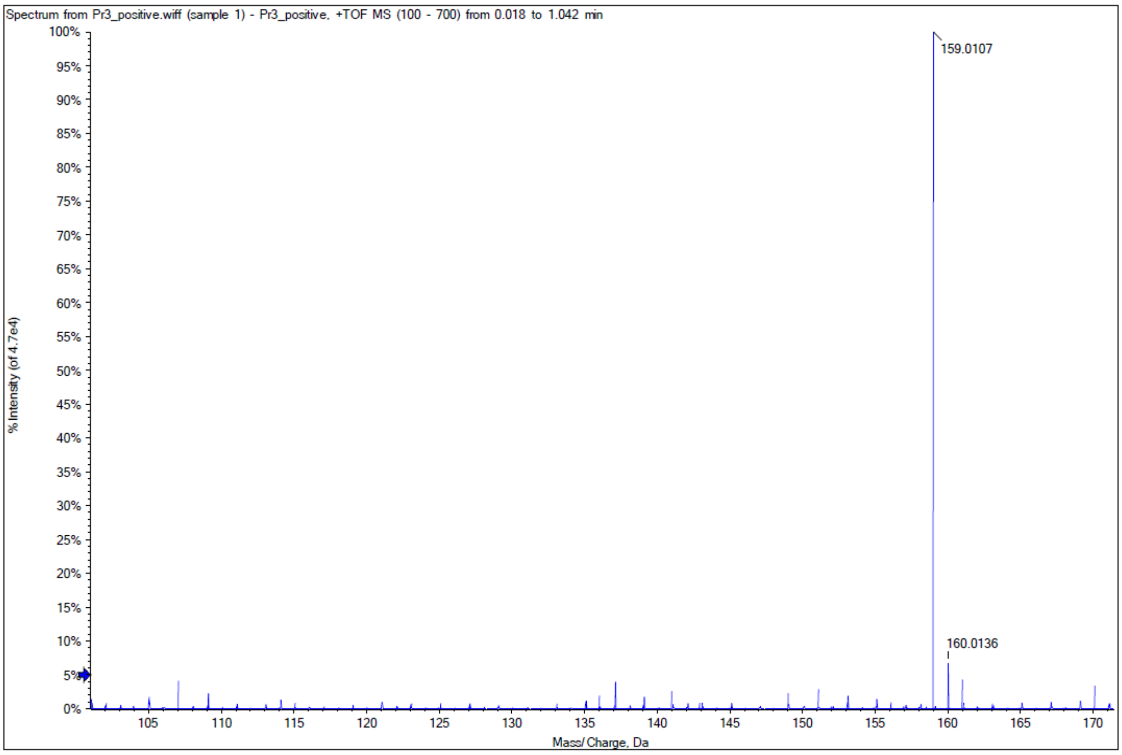

Figure S7. ESI-MS(+) of benzenesulphonic acid radical cation standard with molecular formula $C_6H_5O_3SH_2$ ($M_{BA}H_2$, m/z – 159.0107)

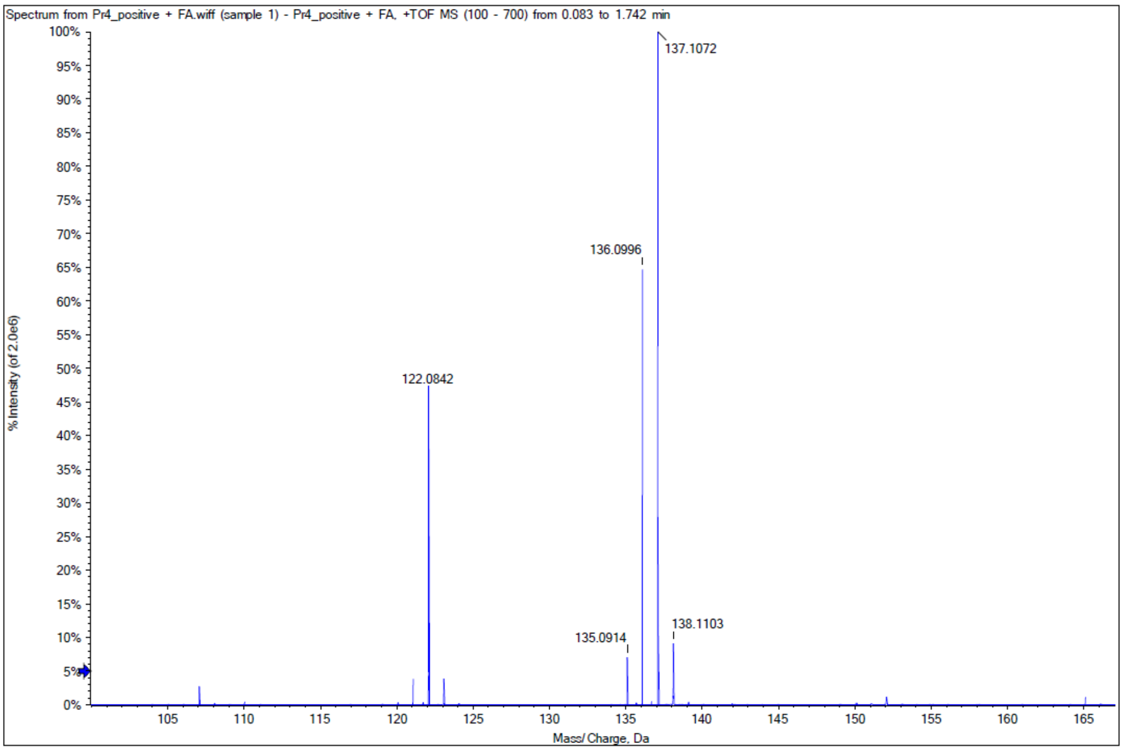

Figure S8. ESI-MS(+) of N,N-dimethyl-p-phenylenediamine radical cation standard with molecular formula, $C_8H_{11}N_2H_2$ (M_DH, m/z – 137.1072)

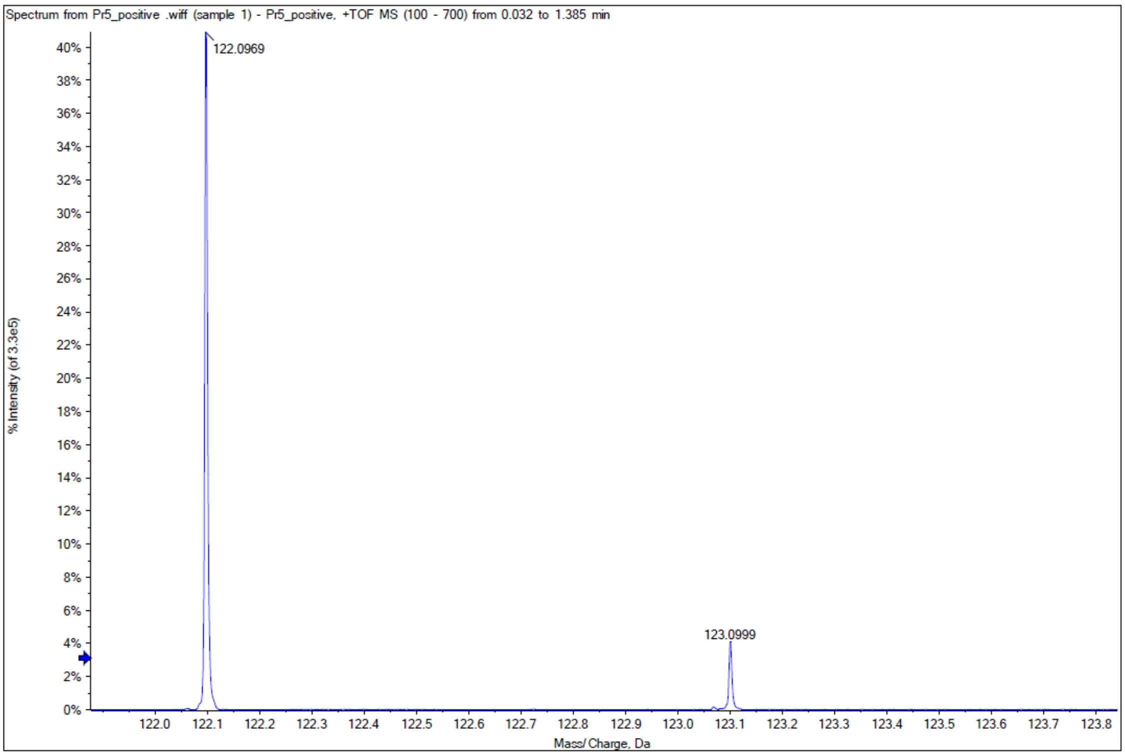

Figure S9. ESI-MS(+) of N,N-dimethylbenzenamine radical cation standard with molecular formula $C_8H_{10}NH_2$ ($M_{DB}H_2$, m/z = 122.0969)

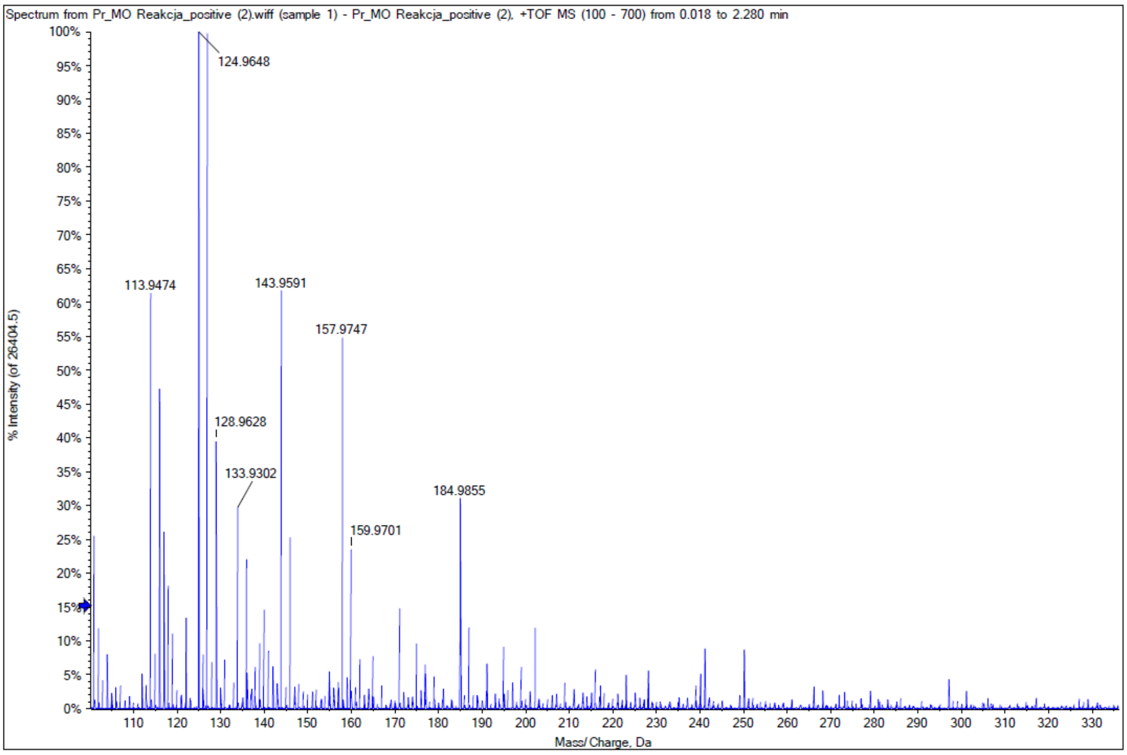

Figure S10. ESI-MS(+) of MO reaction mixture, Mass/Charge – 100-700 [Da]

POSITIVE – ION MODE + FA (Formic acid)

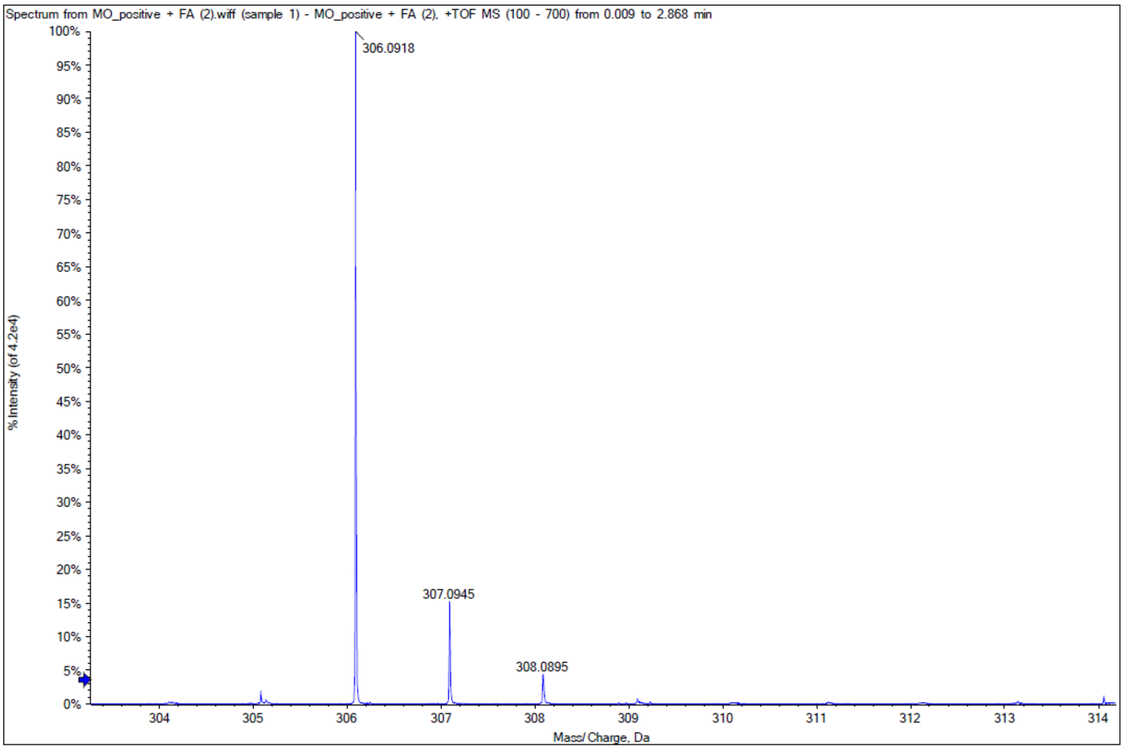

Figure S11. ESI-MS(+) + FA of MO standard with molecular formula $C_{14}H_{16}N_3SO_3$ ($M_{MO}H_2$, m/z=306.0918)

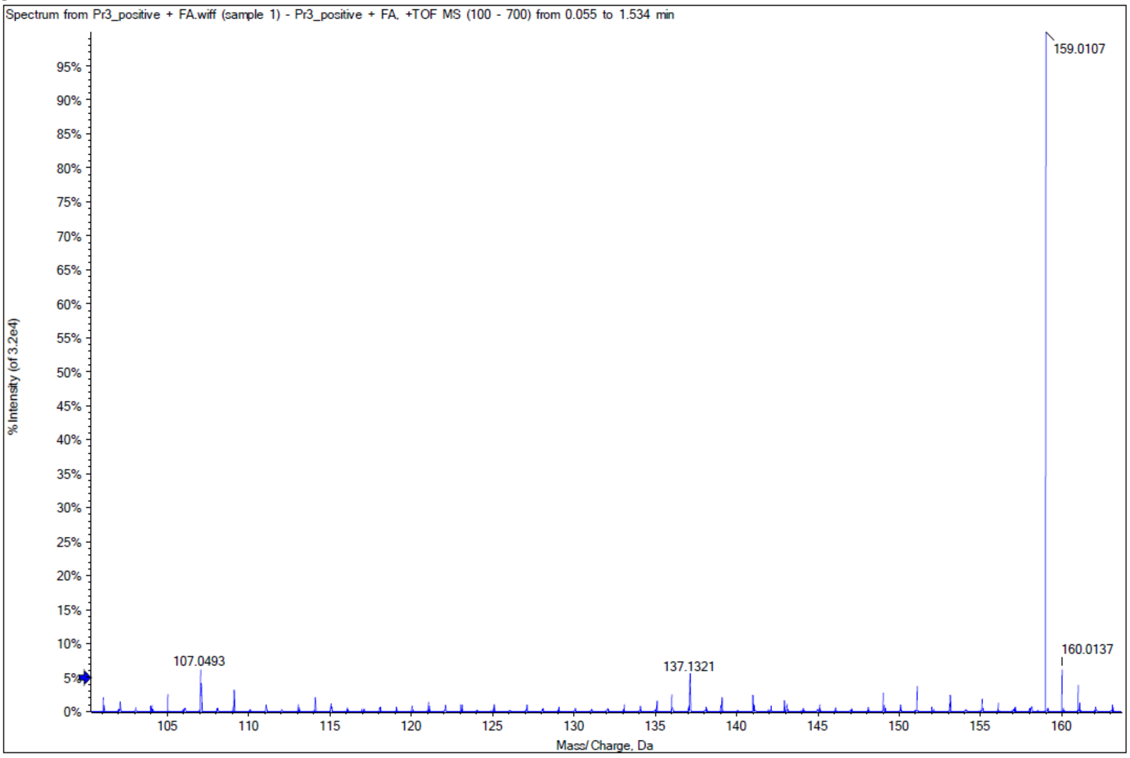

Figure S12. ESI-MS(+) + FA of benzenesulphonic acid radical cation standard with molecular formula $C_6H_5O_3SH_2$ ($M_{BA}H_2$, m/z – 159.0107)

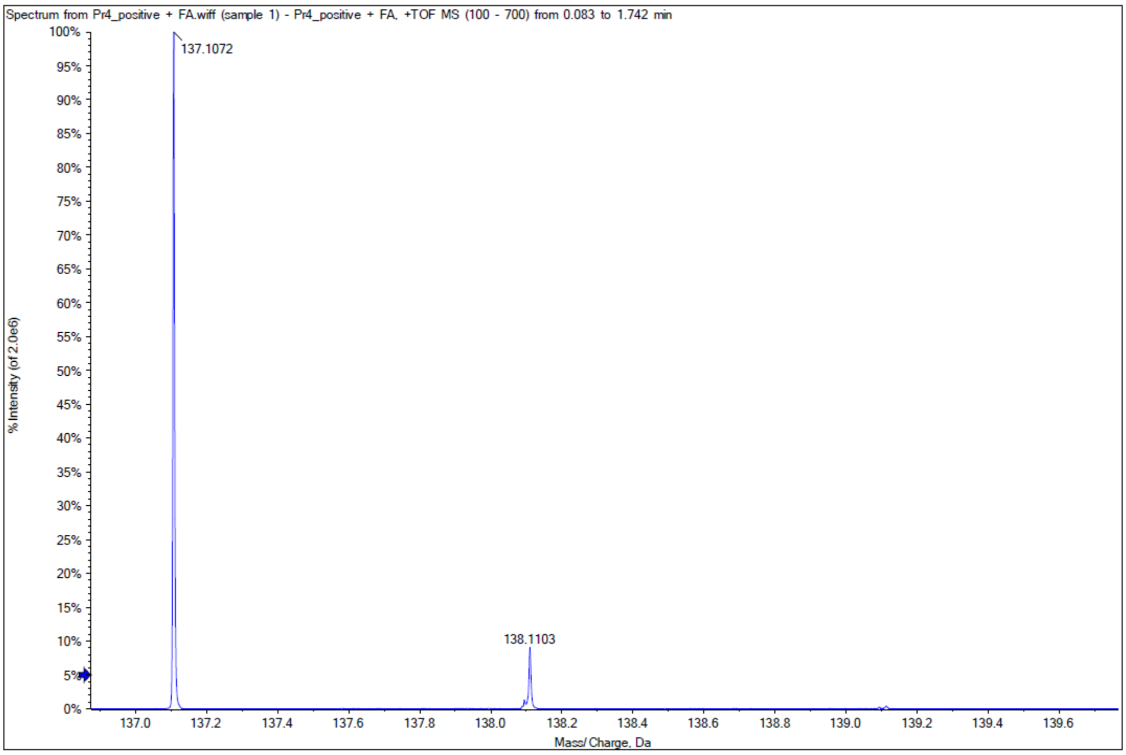

Figure S13. ESI-MS(+) + FA of N,N-dimethyl-p-phenylenediamine radical cation standard with molecular formula, $C_8H_{11}N_2H_2$ (M_DH, m/z – 137.1072)

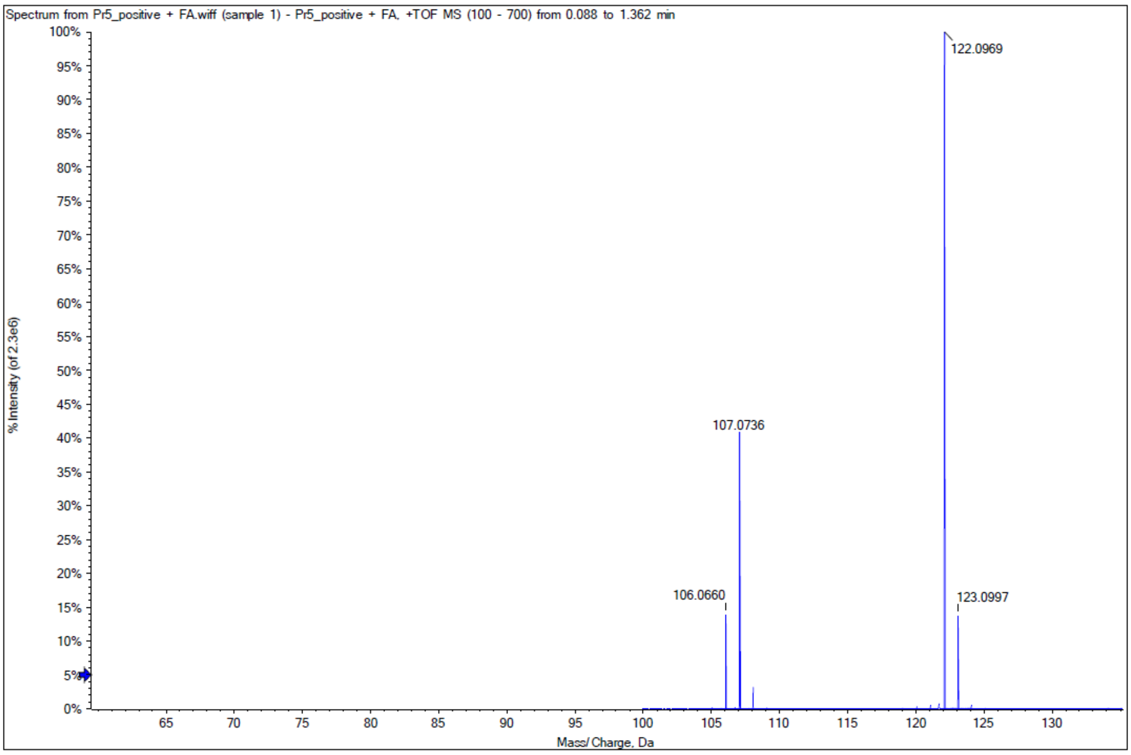

Figure S14. ESI-MS(+) + FA of N,N-dimethylbenzenamine radical cation standard with molecular formula $C_8H_{10}NH_2$ ($M_{DB}H_2$, m/z = 122.0969)

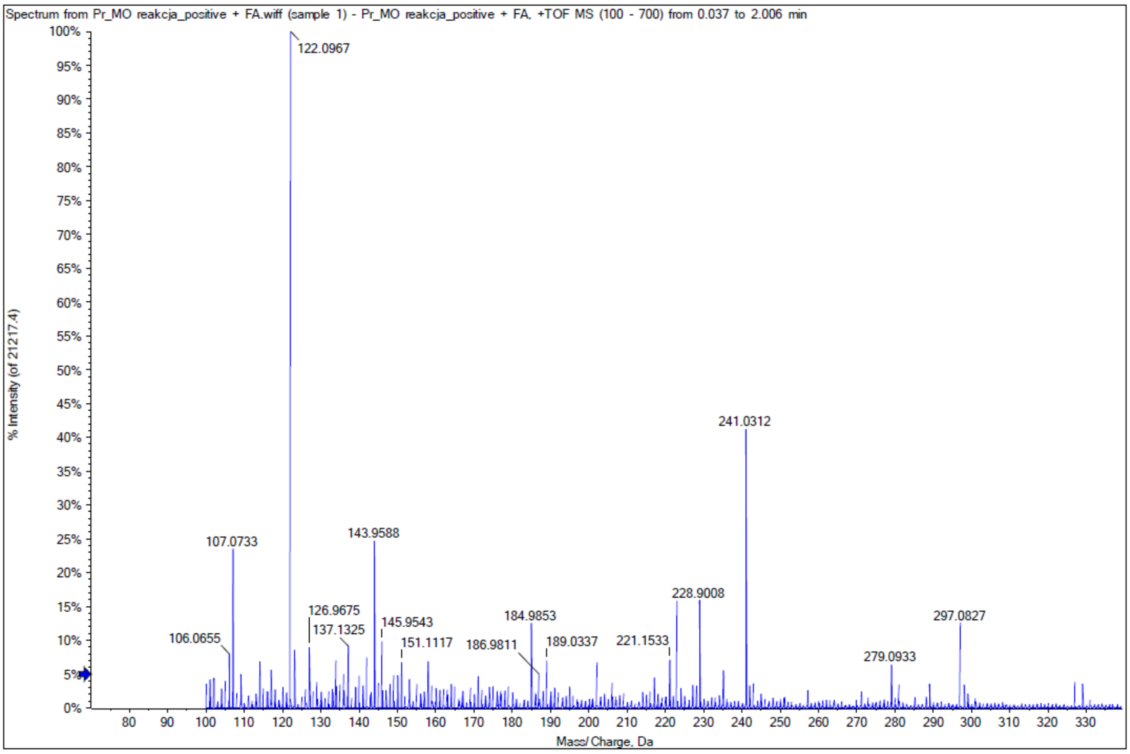

Figure S15. ESI-MS(+) + FA of MO reaction mixture, Mass/Charge – 100-700 [Da]
